# Pyrophosphate‐fructose 6‐phosphate 1‐phosphotransferase (PFP1) regulates starch biosynthesis and seed development via heterotetramer formation in rice (*Oryza sativa* L.)

**DOI:** 10.1111/pbi.13173

**Published:** 2019-06-14

**Authors:** Chen Chen, Bingshu He, Xingxun Liu, Xiaoding Ma, Yujie Liu, Hong‐Yan Yao, Peng Zhang, Junliang Yin, Xin Wei, Hee‐Jong Koh, Chen Yang, Hong‐Wei Xue, Zhengwu Fang, Yongli Qiao

**Affiliations:** ^1^ College of Agriculture Yangtze University Jingzhou China; ^2^ Shanghai Key Laboratory of Plant Molecular Sciences College of Life Sciences Shanghai Normal University Shanghai China; ^3^ Institute of Industrial Crops Songyuan Academy of Agricultural Sciences Songyuan China; ^4^ Key Laboratory of Grains and Oils Quality Control and Processing College of Food Science and Engineering Nanjing University of Finance and Economics Nanjing China; ^5^ National Key Facility for Crop Gene Resources and Genetic Improvement Institute of Crop Science Chinese Academy of Agricultural Sciences Beijing China; ^6^ CAS‐Key Laboratory of Synthetic Biology CAS Center for Excellence in Molecular Plant Sciences Shanghai Institute of Plant Physiology and Ecology Chinese Academy of Sciences Shanghai China; ^7^ National Key Laboratory of Plant Molecular Genetics CAS Center for Excellence in Molecular Plant Sciences Shanghai Institute of Plant Physiology and Ecology Chinese Academy of Sciences Shanghai China; ^8^ School of Agriculture and Biology Shanghai Jiao Tong University Shanghai China; ^9^ Department of Plant Science College of Agriculture and Life Sciences, and Plant Genomics and Breeding Institute Seoul National University Seoul Korea

**Keywords:** floury endosperm, rice, pyrophosphate‐fructose 6‐phosphate 1‐phosphotransferase (PFP1), map‐based cloning, starch synthesis

## Abstract

Pyrophosphate‐fructose 6‐phosphate 1‐phosphotransferase (PFP1) reversibly converts fructose 6‐phosphate and pyrophosphate to fructose 1, 6‐bisphosphate and orthophosphate during glycolysis, and has diverse functions in plants. However, mechanisms underlying the regulation of starch metabolism by PFP1 remain elusive. This study addressed the function of PFP1 in rice floury endosperm and defective grain filling. Compared with the wild type, *pfp1‐3* exhibited remarkably low grain weight and starch content, significantly increased protein and lipid content, and altered starch physicochemical properties and changes in embryo development. Map‐based cloning revealed that *pfp1‐3* is a novel allele and encodes the regulatory β‐subunit of PFP1 (PFP1β). Measurement of nicotinamide adenine dinucleotide (NAD+) showed that mutation of *PFP1β* markedly decreased its enzyme activity. PFP1β and three of four putative catalytic α‐subunits of PFP1, PFP1α1, PFP1α2, and PFP1α4, interacted with each other to form a heterotetramer. Additionally, PFP1β, PFP1α1 and PFP1α2 also formed homodimers. Furthermore, transcriptome analysis revealed that mutation of *PFP1β* significantly altered expression of many essential enzymes in starch biosynthesis pathways. Concentrations of multiple lipid and glycolytic intermediates and trehalose metabolites were elevated in *pfp1‐3* endosperm, indicating that PFP1 modulates endosperm metabolism, potentially through reversible adjustments to metabolic fluxes. Taken together, these findings provide new insights into seed endosperm development and starch biosynthesis and will help in the breeding of rice cultivars with higher grain yield and quality.

## Introduction

The endosperm occupies most of the space in a mature rice (*Oryza sativa* L.) seed and supplies energy and resources for seed germination and seedling growth. Among all metabolic substances in rice endosperm, starch is the most predominant storage substance, accounting for approximately 90% of the dry seed weight, and providing up to 80% of the calories. Starch properties of rice grains affect the quality of cooked rice. More importantly, starch is the main source of carbohydrates and is an important part of a healthy human diet (Hannah and James, 2008). There is a prodigious demand for improvement of the starch properties of rice grains to meet increases in standards of living.

Starch is a polymeric carbohydrate comprising of a huge number of glucose units joined via glycosidic bonds. Amylose and amylopectin are components of starch; the former comprises glucose moieties linked together by α‐1, 4‐glycosidic bonds, whereas the latter comprises glucose moieties connected via α‐1, 6‐glycosidic bonds. Both amylose and amylopectin are synthesized in amyloplasts (Smith, 1999). Starch synthesis in seeds is a complex and coordinated process involving a series of key biosynthetic enzymes. The disruption of genes encoding these enzymes alters the appearance of the seeds and changes the characteristics of seed endosperm starch. For instance, mutations in the gene encoding AGPase subunit result in abnormal starch granules (SG) and considerably decrease starch content (Lee *et al*., 2007; Vandeputtea *et al*., 2003). Mutations in the *GBSSI* gene affect amylose biosynthesis and produce waxy endosperm (Sato *et al*., 2002). Knockout mutation of *OsSSIIIa* leads to a floury endosperm with a white core and affects the physiochemical properties of starches (Ryoo *et al*., 2007). Other starch biosynthesis genes in rice, including *OsPUL*,* OsISA1*,* OsSSI*,* OsBEI* and *OsBEIIb* (Fujita *et al*., 2006; Kubo *et al*., 1999; Li *et al*., 2009; Satoh *et al*., 2003), regulate the structure and physicochemical properties of starch in rice grain.

In addition to starch synthesis enzymes, other genes indirectly affect starch biosynthesis, resulting in an abnormal endosperm. For instance, plastidic pyruvate kinase (OsPK2) and pyruvate orthophosphate dikinase (PPDK) in the glycolytic pathway influence starch granule formation and grain filling (Cai *et al*., 2018; Kang *et al*., 2005). Additionally, PDIL1‐1 (disulphide isomerase‐like enzyme), which is distributed to the endoplasmic reticulum, regulates starch biosynthesis and produces small grains with a floury endosperm (Han *et al*., 2012). The pre‐mRNA processing protein, Du1, regulates starch synthesis by promoting the splicing of *Wxb* (Zeng *et al*., 2007). The nuclear localized TPR‐binding protein FLO2 influences starch biosynthesis, possibly via interactions with transcription factors, such as basic helix‐loop‐helix (bHLH) proteins, to positively affect the expression of genes involved in starch synthesis (Qiao *et al*., 2010; She *et al*., 2010). The carbohydrate‐binding domain (CBM) containing protein, FLO6, regulates starch biosynthesis and compound starch granule formation through interacting with ISA1 (Peng *et al*., 2014). Other factors including FLO7, FLO13, FSE1, DU3, OsZIP58, RSR1, SSG4 and SSG6 have also been demonstrated to affect starch biosynthesis and endosperm development in rice (Fu and Xue, 2010; Hu *et al*., 2018; Isshiki *et al*., 2008; Long *et al*., 2018; Matsushima *et al*., 2014, 2016; Wang *et al*., 2013; Zhang *et al*., 2016). These results suggest that the genetic basis of starch biosynthesis and seed development is complex and involves many starch biosynthesis enzymes and regulatory factors. Therefore, the identification and functional analysis of additional endosperm mutants is essential for further elucidating the process of starch biosynthesis in rice.

PFP1 catalyses the phosphorylation of fructose‐6‐phosphate (F6P) to fructose‐1,6‐bisphosphate (FBP) in the glycolytic pathway, and the dephosphorylation of FBP to F6P in the gluconeogenic direction, and is localized solely to the cytosol (Nielsen *et al*., 2004; Plaxton, 1996). Plant PFPs are expressed in various tissues and developmental periods (Stitt, 1990). In plant cells, PFP exists mainly as a heterotetramer, comprising two catalytic β‐ and two regulatory α‐subunits; however, copy number variations of genes encoding these subunits have been identified in plants (Wong *et al*., 1990). For example, each subunit (α and β) is encoded by a single protein in castor and potato (Carlisle *et al*., 1990; Todd *et al*., 1995) and two proteins in *Arabidopsis* (Lim *et al*., 2014), while four genes encoding each subunit have been annotated in the Rice Annotation Project Database (RAP‐DB; Duan *et al*., 2016).

However, various studies have reported direct and indirect actions of PFP enzymes in both glycolysis and gluconeogenesis (Stitt, 1990; ). In sugarcane, PFP activity is negatively correlated with sucrose accumulation in stalks (Whittaker and Botha, 1999). In sweet pepper, PFP activity is inversely correlated with hexose content in the fruit (Nielsen *et al*., 1991). Changes in PFP activity affect sugar and acid contents as well as sugar ingredients in strawberries fruits (Basson *et al*., 2010). Moreover, PFPs are involved in stress responses under various conditions such as anoxia, wounding, phosphate starvation, high salt and dehydration (Huang *et al*., 2008; Lim *et al*., 2014; Mertens, 1991; Van Schaftingen and Hers, 2010). Thus, PFPs are involved in diverse physiological processes, and their precise functions and significance remain unknown. Recently, *PFP* genes were characterized during endosperm development in maize and rice (Duan *et al*., 2016; Guo *et al*., 2012). However, much less is known about the dynamics and regulatory network of *PFP* genes during seed development.

In this study, we identified a defective grain filling and floury endosperm mutant, *pfp1‐3*. Map‐based cloning revealed that *pfp1‐3* is a novel mutant allele of the gene encoding the β‐subunit of PFP1 (*PFP1β*). Analysis of the *PFP1β* molecular mechanism, transcriptome and starch physicochemical properties revealed that rice PFP1 proteins form heterotetramers; disruption of the *PFP1β* subunit affects starch biosynthesis, starch granule formation and grain weight, and perturbs the lipid and glycolytic pathway and starch biosynthesis‐related gene expression during endosperm development. Our work presents an in‐depth analysis of *PFP1*. These data provide new insights into how rice quality can be improved via genetic approach and increase our understanding of the molecular mechanisms of grain development.

## Results

### Phenotypic characterization of the *pfp1‐3* mutant

**Figure 1:**
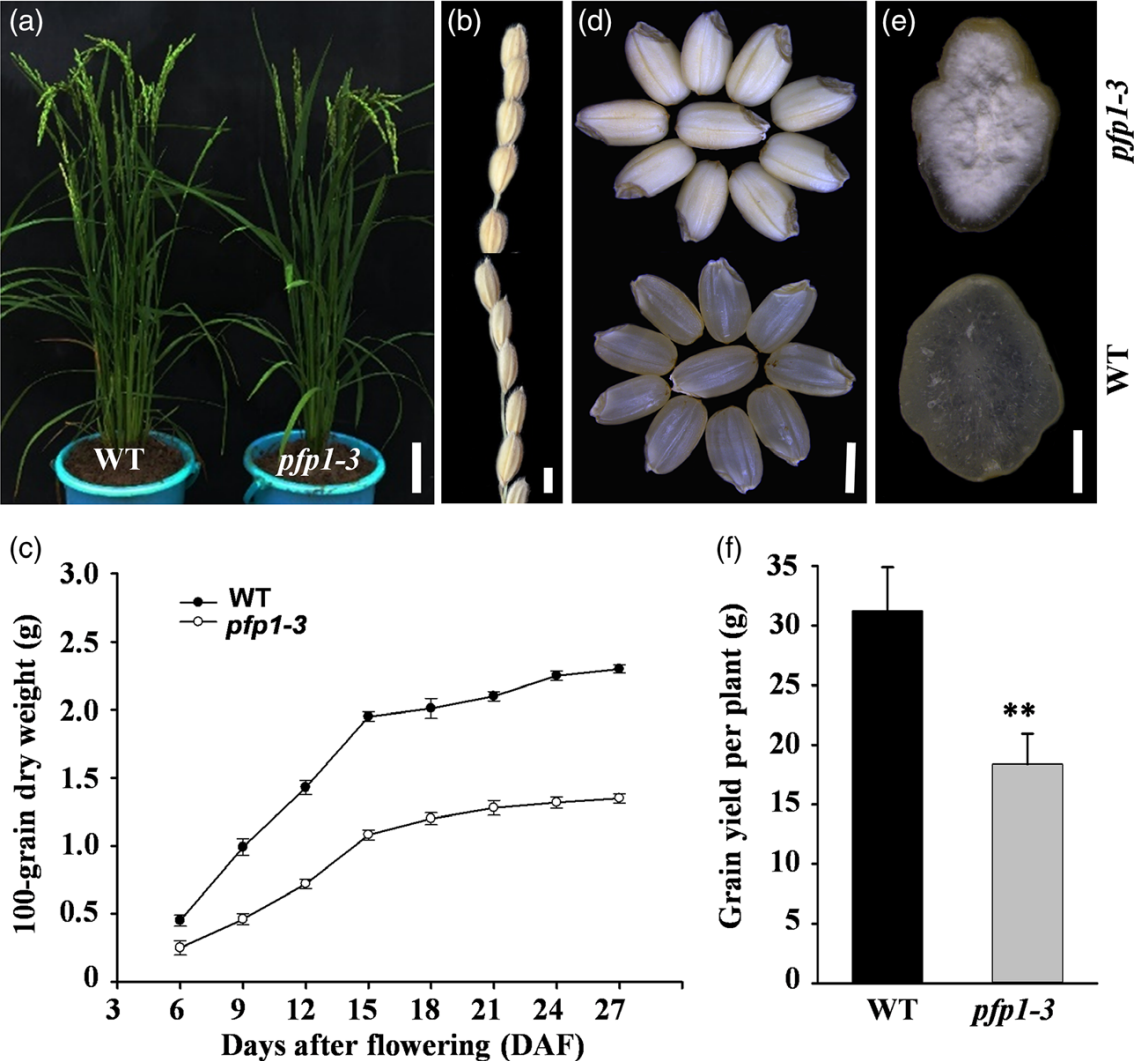
Phenotypic characterization of *pfp1‐3* mutant and wild‐type rice plants. (a) Whole plant phenotype of the *pfp1‐3* mutant and the wild type. (b) Spikelets of the primary inflorescence branch at full maturation. (c) Grain‐filling process. (d) Morphology of dehusked seeds. (e) Horizontal sections of polished seed endosperms. The floury inner region of the endosperm is evident in the *pfp1‐3* mutant. (f) Grain yield per plant in natural paddy field conditions. Data represent mean ± standard error (SE) of three biological replicates. Asterisks represent significant differences between *pfp1‐3* mutant and wild‐type plants (**, *P *<* *0.01; Student's *t*‐test). Scale bars, 10 cm in (a); 3 mm in (b, d); 1 mm in (e).

To identify new factors regulating endosperm development, we identified a defective endosperm mutant of the *japonica* rice cultivar Hwacheong induced by *N*‐methyl‐*N*‐nitrosourea (MNU; Duan *et al*., 2016). The *pfp1‐3* mutant showed no visibly abnormal phenotype during the seedling and tillering stage compared with wild‐type plants (Figure 1a). Phenotype of mature kernels of *pfp1‐3* was also similar to that of wild‐type kernels (Figure 1b). After flowering, the *pfp1‐3* mutant exhibited a significantly slower grain‐filling rate during seed development (Figure 1c). Compared with the normal transparent endosperm in wild‐type grains, husked *pfp1‐3* mutant grains exhibited an opaque and brittle endosperm, with a floury appearance (Figure 1d). Cross‐sectional analysis of rice grains further revealed that the central region of *pfp1‐3* mutant grains was floury white, while the exterior portion maintained a normal transparent appearance (Figure 1e).

Next, we investigated the effect of *PFP1* mutation on grain shape and yield (Figure S1). The length and width of grains were mostly comparable between the wild type and *pfp1‐3* mutant (Figure S1a, b). However, the 1000‐grain weight of the *pfp1‐3* mutant was significantly reduced (34.9%) compared with the wild type (Figure S1c). In addition to differences in the number of panicles per plant (Figure S1d) and plant height (Figure S1e), the *pfp1‐3* mutant displayed a statistically substantial decrease in grain yield per plant (41.2%; Figure 1f) in the paddy field. No significant differences were detected in other agronomic characters such as panicle length, grain number per panicle and heading date (Figure S1f–h).

### Compound starch granule formation is defective in the *pfp1‐3* mutant

**Figure 2:**
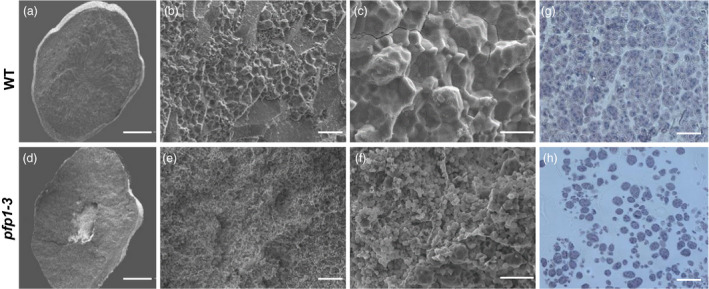
Abnormal endosperm development in *pfp1‐3* seeds. (a–f) Scanning electron microscope analysis of transverse sections of wild‐type seeds (a–c) and *pfp1‐3* mutant seeds (d–f). Starch granules in *pfp1‐3* mutant seeds are irregularly shaped and loosely packed than those in wild‐type seeds. (g, h) Semi‐thin sections of wild‐type (g) and *pfp1‐3* (h) centre endosperm at 15 days after flowering (DAF). Scale bars 1 mm in (a, d); 30 μm in (b, e); 10 μm in (c, f); 50 μm in (g, h).

To confirm changes in the morphology of starch granules in *pfp1‐3* grains, we examined cross sections of polished rice seeds using a scanning electron microscope (SEM). The *pfp1‐3* mutant endosperm showed small, spherical and loosely packed starch grains with large air spaces (Figure 2a–c), whereas wild‐type endosperm contained large, irregularly polyhedral and densely packed starch granules (Figure 2d–f). Moreover, the size of starch granules in *pfp1‐3* mutant seeds varied greatly. These data indicated that the insufficient accumulation of starch granules in *pfp1‐3* mutant resulted in the floury endosperm. Next, we analysed and compared semi‐thin sections of wild‐type and *pfp1‐3* mutant endosperms at 15 days after flowering (DAF). The wild‐type endosperm contained many sharp‐edged, easily separable and polyhedral granules in the central region (Figure 2g), whereas cells in the *pfp1‐3* endosperm had a large number of scattered, immature and small starch granules (Figure 2h).

### Map‐based cloning and complementation of the *PFP1* gene

For genetic analysis of the *PFP1* gene, we crossed the *pfp1‐3* mutant (*japonica*) with the *japonica* cultivar ZH11 and *indica* cultivar Dular, and examined the husked grains of F_1_ and F_2_ progenies derived from both crosses. All F_1_ seeds displayed the wild‐type phenotype, whereas F_2_ seeds exhibited a 3:1 (normal: floury) segregation ratio (Table S1), suggesting that the floury endosperm phenotype was governed by a single nuclear recessive gene.

**Figure 3:**
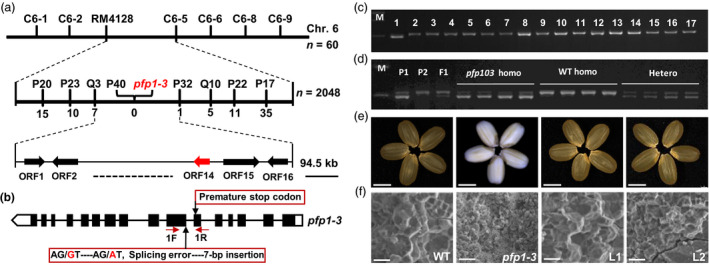
Positional cloning of the *pfp1‐3* mutation and complementation testing. (a) Fine mapping of the *PFP1* locus. The *PFP1* locus (red arrowhead) was mapped to a 94.5‐kb region between markers Q3 and P32 on chromosome 6 (Chr. 6), which contained 16 predicted open reading frames. Marker names and number of recombinants are shown above the map. (b) Schematic representation of the *PFP1* gene structure showing the point mutation site of *pfp1‐3*. Mutant alleles of the *PFP1* gene included a nucleotide substitution that generated an alternative splicing site, resulting in a 7‐bp insertion and premature stop codon (closed red rectangle). Black boxes indicate exons. White boxes indicate non‐coding regions. Lines represent introns. (c) PCR‐based confirmation of splice variants of *PFP1* in the *pfp1‐3* mutant (lane 1) and 16 wild‐type rice cultivars (lanes 2–17). (d) Co‐segregation analysis of the splicing error mutation with the *pfp1‐3* mutant phenotype in the *pfp1‐3 *×** ZH11 F_2_ population based on PCR product size. P1, *pfp1‐3* mutant; P2, ZH11; M, DNA marker. (e, f) Functional complementation of the *PFP1* gene completely rescued normal grain appearance (e) and restored normal starch granule arrangement (f). Representative images of whole seeds (e) and scanning electron microscope images of transverse sections of seeds (f) of wild‐type, *pfp1‐3* and *pfp1‐3* complementation lines (L1 and L2). Scale bars represent 3 mm (e) or 10 μm (f).

To elucidate the molecular mechanism underlying the *pfp1‐3* phenotype, a map‐based cloning strategy was used to isolate the *PFP1* gene. Sixty plants showing the *pfp1‐3* phenotype were selected from the F_2_ progeny of a cross between *pfp1‐3* and Dular. The *PFP1* locus was first localized to the short arm of chromosome 6 between the insertion/deletion (InDel) marker C6‐6 and simple sequence repeat (SSR) marker RM4128 (Table S2). The *PFP1* locus was further delimited to a 94.5‐kb region, flanked by the InDel markers Q3 and P32. No recombinant line was obtained for the STS marker, suggesting that P40 co‐segregates with the *PFP1* gene (Figure 3a). This region contains 16 open reading frames (http://rice.plantbiology.msu.edu/; Table S3). DNA sequence analysis uncovered a single nucleotide substitution of guanine (G) to alanine (A) in the splice site of the 10th exon of *PFP1* (*LOC_Os06g13810*), resulting in a 7‐bp insertion and premature stop codon in the *pfp1‐3* mutant (Figures 3b, S2).

To examine whether the G‐to‐A substitution in the *PFP1* gene was a natural variation present in other cultivars, we genotyped 16 typical *indica* and *japonica* rice cultivars using a derived cleaved amplified polymorphic sequence (dCAPS) marker. All 16 cultivars revealed undigested fragments corresponding to the wild‐type *Pfp1* allele (Figure 3c, Tables S2 and S4). Furthermore, genotypes associated with the dCAPS marker co‐segregated with the matching phenotypes in the F_2_ population (Figure 3d).

To confirm whether *LOC_Os06g13810* corresponded to the candidate gene *PFP1*, we performed a complementation assay. A vector containing the *PFP1* coding sequence under the control of the ubiquitin promoter (*ubq::PFP1*) was introduced into the *pfp1‐3* mutant, and transgenic lines were identified by PCR analysis. Two independent transgenic lines showed the complete rescue of developmentally abnormal endosperm, including compound starch granule arrangement and grain appearance (Figure 3e, f). Overall, we conclude that *LOC_Os06g13810* is the gene responsible for the *pfp1‐3* phenotype. Other mutant alleles of *LOC_Os06g13810* (*pfp1‐1* and *pfp1‐2*) result in opaque grains with a floury endosperm (Duan *et al*., 2016).

### Disruption of *PFP1* exhibits a reduction in enzyme activity and seed storage substances

**Figure 4:**
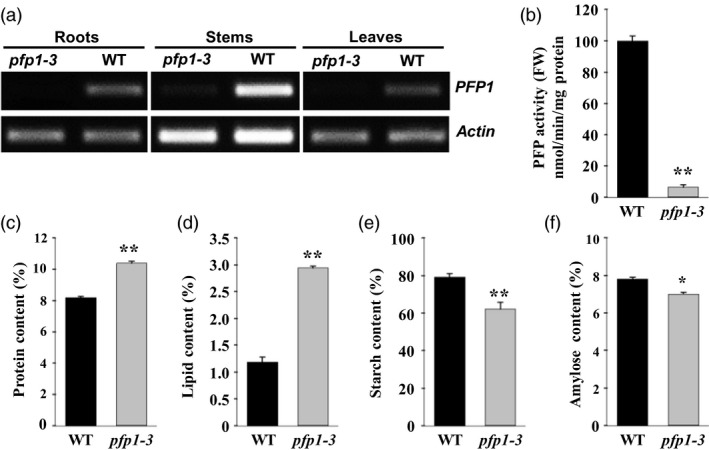
Molecular and phenotypic characterization of wild‐type and *pfp1‐3* mutant plants. (a) Expression of *PFP1* in the root, stem and leaf tissues. (b) PFP1 enzyme activity assays. (c–f) Contents of protein (c), lipid (d), starch (e) and amylose (f) in mutant and wild‐type seeds. Values represent the mean ± SE of three biological replicates. Asterisks represent significant differences between the *pfp1‐3* mutant and wild type (*, *P* < 0.05; **, *P* < 0.01; Student's *t*‐test).

To understand the physiological role of *PFP1*, the expression of *PFP1* was examined in roots, stems and leaves of wild‐type and *pfp1‐3* mutant plants by RT‐PCR analysis. The results revealed that *PFP1* was expressed in wild‐type plants in all organs tested; however, *pfp1‐3* mutant plants showed highly reduced expression of *PFP1* (Figure 4a). Consistent with the gene expression data, PFP1 enzyme activity in *pfp1‐3* mutant plants was 6.3% of that in wild‐type plants (Figure 4b). To determine the reason why seed weight in the *pfp1‐3* mutant was significantly lower than that in the wild type, we investigated several major storage compositions of seeds. The results demonstrated that the seed protein content was significantly higher in the *pfp1‐3* mutant than in the wild type (Figure 4c), and the seed lipid content was simultaneously increased by more than twofold (Figure 4d). However, starch content in the *pfp1‐3* mutant was markedly reduced, and seed amylose content was slightly different between the wild‐type and *pfp1‐3* mutant plants (Figure 4e, f).

### Mutation of the *PFP1* gene alters the physicochemical properties of starch

**Figure 5:**
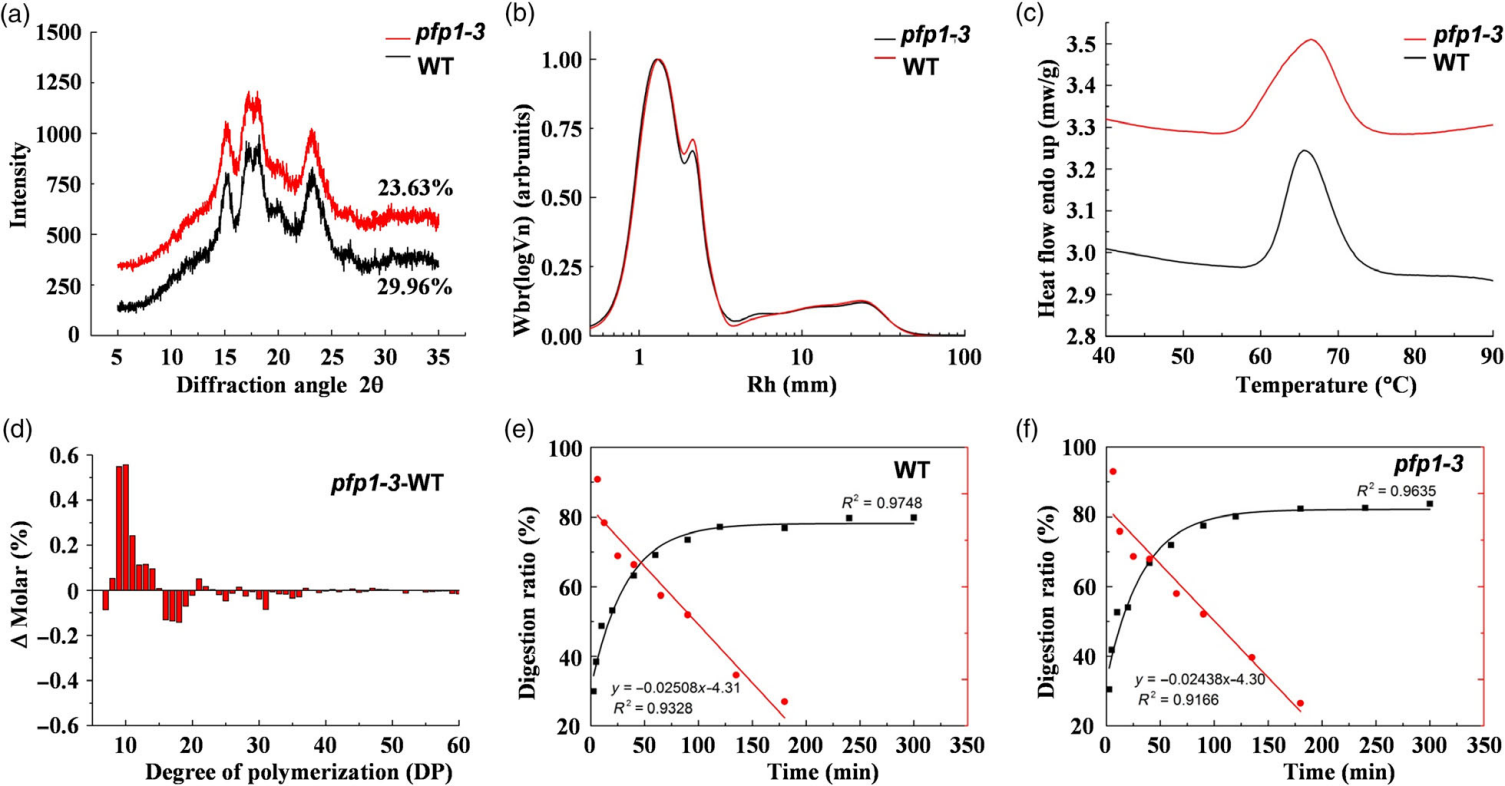
Physicochemical properties of starch in the *pfp1‐3* mutant. (a) X‐ray diffraction patterns of starch granules purified from mature endosperm of the *pfp1‐3* mutant and its wild‐type cultivar, Hwacheong. (b) Size exclusion chromatography (SEC) weight chain length distributions (CLDs) of debranched starches. (c) Differential scanning calorimeter thermograms of *pfp1‐3* mutant and wild‐type starches. (d) Differences in amylopectin chain length distributions between the wild type and *pfp1‐3* mutant. (e, f) Starch digestion curves and LOST plots of endosperm starch in wild‐type seeds (e) and *pfp1‐3* mutant seeds (f).

To further understand the function of PFP1 in starch biosynthesis, we performed wide‐angle X‐ray diffraction analysis using seed endosperm starch. Starch in the endosperm of both *pfp1‐3* mutant and wild‐type grains fitted a typical pattern of A‐type crystals, with robust diffraction peaks at 2θ angle of about 15, 17, 18 and 23, and additional weak peaks at 20 and 27 (Figure 5a). The relative degree of crystallinity was calculated as 29.96% and 23.63% for wild‐type and *pfp1‐3* seed starch, respectively.

We next investigated the molecular structure of starch in *pfp1‐3* and wild‐type seeds using size exclusion chromatography (SEC). The SEC spectra of individual chains were obtained from debranched starch as a function of degree of polymerization (DP). The result showed that both samples had the typical bimodal distribution of amylopectin chains. The first peak, representing short‐chain amylopectin, was similar between *pfp1‐3* and the wild type, whereas the second peak, representing long‐chain amylopectin, was higher in *pfp1‐3* than in the wild type. In addition, no differences were observed between the two samples in the amylose chain, indicating that the branching structure of only long‐chain amylopectin differs between the wild type and *pfp1‐3* mutant (Figure 5b). Moreover, analysis of amylopectin fine structure by high‐performance anion‐exchange chromatography (HPAEC) showed that amylopectin in *pfp1‐3* seeds had more short chains with DP ranging from 8 to 15 and 16 to 50, indicating an increase in the proportion of short outer A chains of amylopectin and a decrease in the proportion of longer B chains (Figure 5d).

Next, we analysed the thermal properties of *pfp1‐3* and wild‐type starch using differential scanning calorimetry (DSC). The *pfp1‐3* starch showed considerably lower gelatinization onset temperature and higher peak temperature than wild‐type starch, with a wider range of gelatinization temperature. The gelatinization conclusion temperature and enthalpy were comparable between the rhizome and bulbil starches, indicating that higher relative degree of crystallinity always leads to higher gelatinization enthalpy (Figure 5c, Table S5).

To determine the digestibility of *pfp1‐3* and wild‐type starches, we analysed the *in vitro* digestion kinetics of cooked rice starch. The results of this analysis related to the molecular structure of rice starch. *In vitro* digestion kinetic profiles showed that the digestion of starch in both samples was rapid in the first hour and almost reached a plateau at the end‐point of digestion. LOS plots of cooked rice starch showed a linear relationship with the rate constant k, indicating that the digestion of fresh cooked starch is a single‐phase process (Figure 5e, f). No substantial difference was found in the speed of starch digestion (*K* value) between *pfp1‐3* and wild‐type samples, while a significant difference was observed in the theoretical proportion of starch digested at the reaction end‐point (Table S6), indicating that *pfp1‐3* starch is easier digestible than wild‐type starch.

### *pfp1‐3* mutant allele affects embryo development in rice

**Figure 6:**
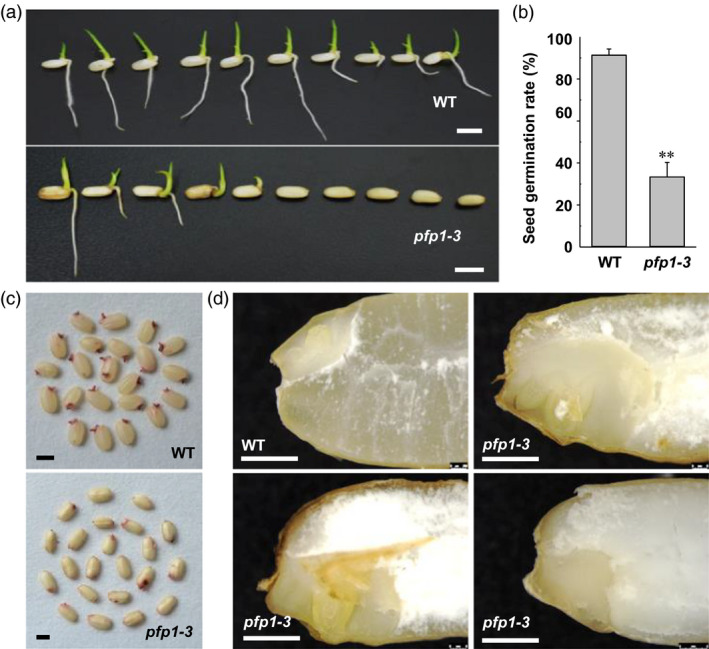
Analysis of seed germination and embryo development in the wild type and *pfp1‐3* mutant. (a) Germination of mature seeds plated on half‐strength MS medium without sugar for 4 days. (b) Seed germination rate. Data represent mean ± SE of three biological replicates, with each replicate containing 30 seeds. Asterisks indicate significant differences between wild‐type and *pfp1‐3* mutant seeds (**, *P *<* *0.01; Student's *t*‐test). (c) Tetrazolium assay of wild‐type and *pfp1‐3* mutant seeds. (d) Vertical sections of imbibed wild‐type and *pfp1‐3* mutant embryos. The *pfp1‐3* mutant seeds contained an abnormal embryo. Scale Bars 6 mm in (a); 3 mm in (c); 1 mm in (d).

To investigate whether seed germination was influenced by the *pfp1‐3* mutation, freshly harvested seeds were placed on half‐strength Murashige and Skoog (1/2MS) medium lacking sugars because the *pfp1‐3* mutant exhibited a low seedling emergence rate under greenhouse and paddy field conditions. Compared with the wild type, the *pfp1‐3* mutant exhibited a significantly low germination rate; more than 67% of the *pfp1‐3* seeds were unable to germinate (no radicle emergence; Figure 6a, b). The low germination rate of *pfp1‐3* seeds led us to investigate seed viability. In the tetrazolium chloride (TTC) staining assay, most of the wild‐type embryos stained deep red, whereas a high percentage of the *pfp1‐3* mutant embryos showed no staining (Figure 6c). To better understand the role of *PFP1* during embryo development, we manually prepared cross sections of imbibed wild‐type and *pfp1‐3* mutant seeds. The results showed that wild‐type seeds formed well‐developed embryos with an intact coleoptile and shoot apical meristem (Figure 6d). By contrast, in approximately 70% of the mature *pfp1‐3* embryos, structures including the radicle and coleoptile were defective (Figure 6d). These data indicate that the *PFP1*β mutation causes abnormal development of embryos.

### PFP1 interacts with three PFP1 α‐subunits to form heteropolymers

**Figure 7:**
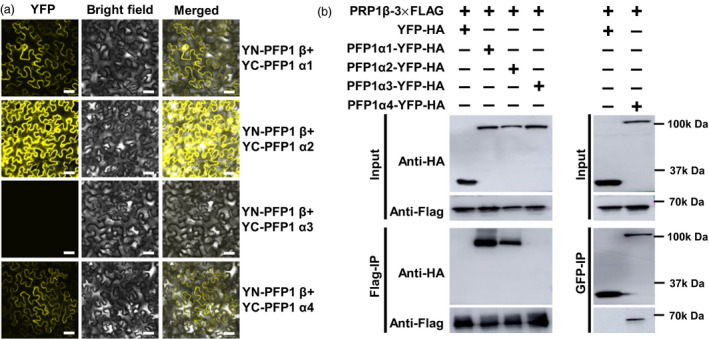
Interactions PFP1β with PFP1α in *planta*. (a) BiFC assay showing interactions of PFP1β with three PFP1α proteins. The BiFC assay was performed in *N. benthamiana* leaves upon *Agrobacterium tumefaciens*‐mediated transient expression. Fluorescence was detected in epidermal cells of infiltrated tissues by confocal microscopy at 48 hpi. Scale Bars, 30 μm. (b) PFP1β and three PFP1α proteins interact in *planta*. Total proteins were extracted from *N. benthamiana* leaves expressing PFP1β–FLAG and PFP1α‐YFP. The immune complexes were pulled down by using anti‐FLAG or GFP agarose gel, and the coprecipitation of PFP1β or PFP1α was detected by Western blotting.

In plants, PFP exists as a heterotetramer, comprising two catalytic β‐subunits and two regulatory α‐subunits, in addition to other multifarious configurations (Guo *et al*., 2012). Although four genes are predicted to encode the putative regulatory α‐subunits, only one gene encoding the catalytic β‐subunit has been annotated in the rice genome (Duan *et al*., 2016). To define the complex formation in rice endosperm, interactions of PFP1β with four PFP1α proteins were examined by BiFC assays. Results showed physical interactions of three putative PFP1α subunits (PFP1α1, PFP1α2 and PFP1α4) with PFP1β (Figure 7a). We further confirmed the interaction of PFP1β with three PFP1α proteins in plant cells. PFP1β–3xFLAG was coexpressed with PFP1α‐YFP‐HA constructs in *N. benthamiana* using *Agro‐*infiltration. Total proteins were extracted from the infiltrated leaves and incubated with anti‐FLAG or GFP resins. PFP1α1‐YFP‐HA, PFP1α2‐YFP‐HA and PFP1α4‐YFP‐HA, but not PFP1α3‐YFP‐HA, YFP‐HA (empty vector), was considerably enriched in the FLAG or GFP precipitates (Figure 7b). These results further suggested that PFP1β interacted with three PFP1α proteins in *planta*. In addition, BiFC assays revealed that PFP1β, PFP1α1 and PFP1α2 interact with themselves and each other to form homodimers and heterodimers, respectively. Moreover, PFP1α4 did not show homodimerization. Furthermore, PFP1α3 did not bind to any PFP1 subunit in *planta*. Thus, PFP1β interacted with some PFP1 subunits by specific binding and formed heterotetramer protein complexes in rice (Figure 5S). Analysis of *PFP1* gene transcription in the wild type and *pfp1‐3* mutant showed that the expression of *PFP1β* was remarkably reduced and that of *PFP1α1*,* PFP1α2* and *PFP1α4* was significantly increased in the *pfp1‐3* mutant. Consistent with BiFC results, expression of *PFP1α3* showed no remarkable differences between the wild type and *pfp1‐3* (Figure S3).

### Mutation of *PFP1β* results in multiple metabolic changes in rice endosperm

To determine the effect of the loss‐of‐function mutation *pfp1‐3* on global gene expression in the developing endosperm, we conducted RNA‐Seq analysis of the developing *pfp1‐3* and wild‐type grains at 3, 6 and 12 days after pollination (DAP). Approximately 81.5% of raw reads from each sample were anchored to the areas annotated as protein‐coding genes (Table S7). A total of 2918, 1594 and 56 genes were upregulated, and 1114, 804 and 161 genes were downregulated in the *pfp1‐3* mutant compared with the wild type (Table S8).

PFP1 is a key enzyme in the glycolysis that catalyses the phosphorylation of F6P. We focused on examining several metabolic processes related to genes and determined whether the starch reduction in the *pfp1‐3* mutant is directly related to the changes in the expression of genes involved in these pathways. The expression of 58 putative enzyme genes associated with glycolytic, pyruvate and fatty acid metabolism was observed in the transcriptome data. Compared with the wild type, most of the genes showed slightly upregulated or similar expression profiles in *pfp1‐3* from 3 to 12 DAF (Figure S6). For example, the expression of levels of five GAPDHs encoding glyceraldehyde‐3‐phosphate dehydrogenases, an enzyme of ~37 kDa that catalyses the sixth step of glycolysis, was slightly increased in *pfp1‐3*.

**Figure 8:**
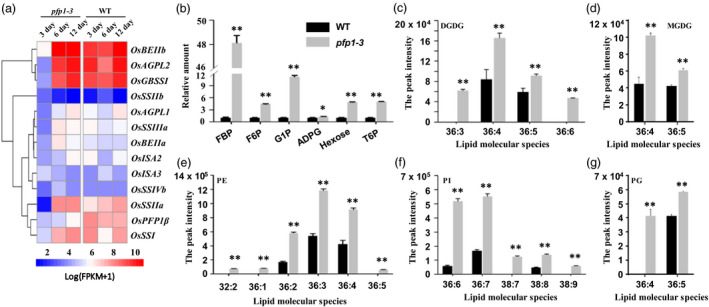
Effect of decreased PFP1 activity on metabolites and gene expression involved in starch biosynthesis. (a) Expression profiles of genes involved in starch biosynthesis. Total RNA was isolated from seeds at 3, 6 and 12 DAF, and subjected to reverse transcription using oligo (dT) primers. Blue and red colours indicate downregulated and upregulated expression levels, respectively, in comparison with expression levels in wild‐type rice plants. (b) Soluble sugars content of glycolytic intermediates and sugar signalling molecule was determined in mature seeds of rice by GC MS analyses. (c–g) The changes of five lipid classes were investigated from individual molecular species in mature rice seeds as revealed by LC‐SI‐MS. Galactolipids including DGDG and MGDG in (c–d). Phospholipids including PE, PG and PI in (e–g). Values represent mean ± SE of two biological replicates. Asterisks represent significant differences between the *pfp1‐3* mutant and wild type (*, *P* < 0.05; **, *P* < 0.01; Student's *t*‐test).

We also investigated the activities of key enzymes in starch biosynthesis. The results showed that the activities of all 12 essential starch synthetic enzymes in the developing *pfp1‐3* endosperm were lower than in the wild type at 3 DAP, but there was no significant change at 6 and 12 DAF (Figure 8a). Therefore, loss of function of the *PFP1* gene had strong effects on the expression of the key starch synthetic genes.

We next evaluated fold‐changes of sugar metabolites by HPLC analysis. The results showed that steady‐state concentrations of multiple glycolytic intermediates and related metabolites were elevated when PFP1 activity was decreased (Figure 8b). FBP showed the most significant increase, with a 48.1‐fold increase in the PFP1‐deficient endosperm. A sugar signalling molecule, trehalose 6‐phosphate (T6P), which regulates starch synthesis through redox activation of AGPase downstream of SnRK1, was also significantly increased (Kolbe *et al*., 2005). HPLC analysis further revealed that ADP‐glucose steady‐state levels were comparable to those of other glycolytic intermediates in the PFP1‐deficient endosperm; however, its levels in the wild type were too low to assign statistical significance. Overall, these results suggested that PFP1 impairs the sugar transport in the PFP1‐deficient endosperm.

We further determined the lipid metabolites in mature rice seeds by LC‐SI‐MS. Nine lipid classes and their molecular species were profiled, including phosphatidic acid (PA), phosphatidylcholine (PC), lysoPC, phosphatidylethanolamine (PE), lysoPE, phosphatidylglycerol (PG), phosphatidylinositol (PI), digalactosyldiacylglycerol (DGDG) and monogalactosyldiacylglycerol (MGDG) (Figure 8c‐g, Figure S7). The content of six different lipid classes (PA, PE, PG, PI, MGDG and DGDG) in all detected molecular species showed significantly increased in *pfp1‐3* relative to the wild type, but the content of LPE exhibited markedly decreased level in *pfp1‐3*. For PC and LPC levels, each molecular species showed inconsistent trends, whereby increase and decrease levels have been observed between *pfp1‐3* and wild type. These results suggested that PFP1 also perturbs the levels of lipid metabolites in the PFP1‐deficient endosperm.

## Discussion

### PFP1β encodes the regulatory β‐subunit in rice

Manipulating starch biosynthesis in rice grains has long been pursued as a way of improving rice grain quality. Recent research indicated that many rice mutants with changes in starch biosynthesis have an opaque endosperm. These mutants constitute valuable genetic resources for unravelling the mechanisms of endosperm nutrition and development and starch biosynthesis (Cai *et al*., 2018), which have heretofore remained poor understood. In this study, we identified the *pfp1‐3* mutant, which exhibited a floury endosperm feature in rice. Positional cloning revealed that *pfp1‐3* is a novel allele of *PFP1β*, which is reported to affect carbon metabolism during grain development in rice (Duan *et al*., 2016). Nevertheless, the mechanism underlying the role of PFP1 in starch biosynthesis in rice has not been reported.

Pyrophosphate‐fructose 6‐phosphate is an enzyme of carbohydrate metabolism in plants and some bacteria that catalyse reversible reactions between F6P and F‐1,6‐BP, a rate‐limiting step in the regulation of primary carbohydrate metabolic flux towards glycolysis and gluconeogenesis (Nielsen *et al*., 2004). PFP1s have diverse functions in plants, including in glycolysis, gluconeogenesis, sugar accumulation, growth development and various stress responses (Huang *et al*., 2008; Lim *et al*., 2007, 2009; Mertens, 1991). PFP1α is also co‐induced with heat shock proteins (Hsps) that regulate opaque endosperm development in maize, which is associated with PFP1α enzyme activity (Guo *et al*., 2012). This is consistent with PFP1β functions in rice. In this study, decreased PFP1β activity resulted in a floury endosperm and affected grain filling, starch physicochemical properties and endosperm nutritional quality (Figures 1, 5 and 8a), indicating that rice endosperm PFP1β is involved in the synthesis of the normal components of storage compounds to ensure that mature seeds have the hard, transparent character associated with good grain quality.

### PFP1β interacts with three of four PFP1α subunits to form heteropolymers

Plant PFPs consisted of α‐ and β‐subunits, and are localized exclusively to the cytosol. Moreover, the PFP enzyme complex exists as a α2β2 heterotetramer or β2 homodimer, depending on the plant species (Guo *et al*., 2012). In this study, we cloned and characterized the *PFP1β* gene, which encodes the regulatory β‐subunit of PFP1 in rice. Confocal analysis showed that the green fluorescence signals of PFP‐GFP were distributed solely in the cytosol (Figure S4), which is consistent with a previous study (Duan *et al*., 2016). Both BIFC and Co‐IP assays revealed the interaction of PFP1β with three of four putative PFP1 α‐subunits (PFP1α1, PFP1α2 and PFP1α4) and homodimerization of PFP1β, PFP1α1 and PFP1α2 (Figure 7). These results indicate that PFP1 proteins in rice exist as a heterotetrameric complex, not as a heteropentamer. Additionally, results of BiFC assays showed that yellow fluorescence signals of PFP1α1 and PFP1α2 were localized to the cytoplasm of cells in *Nicotiana benthamiana* (Figure 7). Previous phylogenetic analysis of PFP1 homologous proteins shows that PFP1α3 is closely related to PFP1α4 (Duan *et al*., 2016). However, PFP1α3 is unable to bind to the other four PFP catalytic and regulatory subunits in rice (Duan *et al*., 2016), indicating that PFP1α3 might have a different function or regulate starch biosynthesis through a direct interaction between PFP1β and some other key essential enzymes. This will require further investigation. Taken together, these results suggest that the PFP protein complex is in more complex and diverse in rice than in other plants, probably because of differences in interacting networks and functional redundancy among PFP1 genes in rice.

### PFP1β causes defects in multiple metabolic flux processes

In this study, mutation in *PFP1* significantly reduced PFP expression and activity in the *pfp1‐3* mutant, which markedly altered the expression levels of many genes, such as *AGPL2*,* AGPS1*,* AGPS2b* and *GBSSI* belonging to the starch biosynthesis pathways (Figure 8a, Table S8), indicating PFP1β regulates starch biosynthesis by affecting the expression of these key genes. Similar results were also reported for other floury rice mutants (Duan *et al*., 2016; Peng *et al*., 2014; She *et al*., 2010). More importantly, we found that some genes expressing proteins of the glycolytic pathway or involved in FA biosynthesis had similar expression pattern and their expression levels (e.g. for instance, PFK, PDH, FBPase and KASI) were not dramatically different between *pfp1‐3* and WT (Figure S6).

In addition, metabolite analysis revealed increased steady‐state concentrations of six glycolytic intermediates in the *pfp1‐3* mutant, including F6P and G1P, confirming that PFP1β acts in the glycolytic direction in rice endosperm and that flux through the glycolytic pathway is decreased when the level of PFP1β is reduced (Figure 8b). The increase in T6P that showed as a general regulator of metabolic pathways, together with elevated F6P, suggested that PFP1β alters sugar metabolic flux in the direction of starch biosynthesis (Zhang *et al*., 2009). This is similar to pyruvate phosphate dikinase (PPDK) functions in maize and rice, and PPDK reversibly converts AMP, pyrophosphate and phosphoenolpyruvate to orthophosphate, pyruvate and ATP. Mutations of *PPDK1* and *PPDK2* resulted in essentially doubling of the steady‐state concentration of multiple glycolytic intermediates. The complete PPDK knockout seeds caused an opaque phenotype (Lappe *et al*., 2018). In maize, the requirement for glycolytic flux regulation is associated with hypoxia in maize endosperm in the endosperm (Rolletschek *et al*., 2005). Intriguingly, the PFP1β deficiency results in lipid metabolic perturbation (Figure 8c‐g, Figure S7) and subsequently may change the quality of the endosperm cell as they mature. Collectively, these results suggested that disruption of starch synthesis results in significantly altered metabolites in complex lipids and sugar metabolism, most of the metabolites had an increase levels in *pfp1‐3*, and they might partially compensate for the lack of function of starch during normal growth and development in rice (Lappe *et al*., 2018; Yu *et al*., 2018). However, how starch, sugar and lipid metabolic pathways interact to regulate metabolism and endosperm development in rice requires further investigation.

### PFP1β has pleiotropic effects on starch biosynthesis and embryo development in rice

The *pfp1‐3* mutant exhibited lower seed germination rate and abnormal embryo development (Figure 6a). This may be due to the increased amount of FA in *pfp1‐3* (Figure 4d), which was also observed in an *AtPKp1* mutant of *Arabidopsis* (Baud *et al*., 2007) and an OsPK2 mutant of rice (Cai *et al*., 2018). Thus far, several genes (*OsPK2*,* OsPPDK*,* OsAGPL2* and *PFP1*) have been found to modulate starch biosynthesis and endosperm development (Cai *et al*., 2018; Duan *et al*., 2016; Kang *et al*., 2005). Our data indicate that *PFP1β* and *OsPK2* employ similar mechanisms for starch biosynthesis in rice grains, enzyme activity regulation and heteropolymer formation in rice grains.

A previous study reported that transgenic tobacco plants with reduced PFP expression had no visible developmental phenotype but had major changes in carbon fluxes, suggesting that strongly decreased PFP enzyme activity might be compensated by FBP in the glycolytic pathway (Nielsen and Stitt, 2001). Nevertheless, *PFP1α* or *PFP1β* subunit overexpression resulted in increased PFP1 enzyme activity and a slightly faster growth relative to the wild type. PFP RNAi *Arabidopsis* lines exhibit retarded growth but no detectable changes in their carbon partitioning profiles in leaves (Lim *et al*., 2009). In the present study, PFP1 expression was significantly reduced in the *pfp1‐3* mutant, but this mutant did not display abnormal growth defects except for defects in seed quality and germination rate. In addition, the transcriptional analysis revealed no observable differences between the pfp1‐3 mutant and the wild type in the expression of genes encoding PFKase, FBPase and PFPase, which are involved in the same step as PFP. This observation likely explains why reduced PFP activity is fully compensated for by increased FBP levels. Nevertheless, our data do not rule out the possibility that PFPα subunits compensate for development defects through elevated expression. qPCR analysis showed that three PFPα subunits of genes that interacted with PFP1β were markedly upregulated (Figure S3). Collectively, our results provide new insights into the action of PFP1β in endosperm development and starch synthesis via modulations in endosperm metabolic fluxes. Moreover, these data provide useful information for the future development of rice plants with higher grain yield and quality.

## Experimental procedures

### Plant materials and phenotypic evaluation

The *pfp1‐3* mutant (*Japonica* cv. Hwacheong) was isolated from a MNU (*N*‐methyl‐*N*‐nitrosourea)‐mutagenized population. The parental plants and their F1, F2 progenies were grown under natural conditions at the Experimental Farm of Chinese Academy of Agricultural Sciences (CAAS), Beijing and Sanya, in China.

For agronomic traits, ten randomly chosen plants were evaluated. Grain length and width were determined at the maximal values for each grain using an electronic digital calliper. Grain weight was initially gained by weighing 1000 dry grains. Plants and matured seeds were photographed with a digital camera (D5100, Nikon) and a stereomicroscope (M165FC, Leica microsystems).

### Seed viability and germination assay

The dehulled rice grains from wild type and *pfp1‐3* were soaked in Petri dish with sterile distilled water at 30 °C for 20 h. Subsequently, the seeds were steeped in a 1% TTC solution (2,3,5‐triphenyltetrazolium chloride) at 37 °C for 2 h in the dark and then rinsed with deionized water until the water ran clear. Rice seed was regarded as viable if the embryo entirely stained red or pink, and embryos without colour were considered as non‐viable.

About 100 seeds of *pfp1‐3* mutant and WT were sterilized and put on half‐strength MS medium without sugar (pH 5.8). Seeds were incubated in a growth chamber with 12/12 light/dark cycle at 30 °C. Germination is calculated as the emergence of the radicals through the seed coat. The experiment was performed with three biological replicates.

### Microscopic analysis

Scanning electron microscopy was performed as described previously (Qiao *et al*., 2015). Mature seeds were transversely cut by knife, and cut grains were coated with gold and the mounted specimens were photographed through a Stereoscan Leica Model 440 Scanning Electron Microscope at an accelerating voltage of 10–20 kV.

Semi‐thin section analysis was carried out according to the protocol described previously (Peng *et al*., 2014). The developing seeds aged 15DAF produced by mutant and wild‐type plants were left in fixed solution including 0.1 m phosphate buffer (pH 7.2) with 2% (v/v) glutaraldehyde and 2% (w/v) paraformaldehyde overnight. After dehydration through an ascending series of ethanol, the subsequent morning the tissues were embedded in LR White resin (London Resin, Berkshire, UK, http://www.2spi.com/), followed by sectioning with an ultramicrotome (Leica UC7; http://www.leicamicrosystems.com). Samples (approximately 1 mm in thickness) were stained for 5 s with I2‐KI, subsequently inspected under a light microscope.

### Genetic mapping and rice transformation

For mapping of the *PFP1* gene, we constructed an F2 population derived from a cross between the *pfp1‐3* mutant and Zhonghua 11 (ZH11), and performed recessive class analysis (RCA) using approximately 200 polymorphic SSR and STS markers equally dispersed over the whole rice chromosomes. To fine mapping of *OsPFP1* gene, InDel and SNP markers were developed based on the nucleotide polymorphisms in the corresponding regions between the Nipponbare and Dular. The primer sequences used are shown in Table S1.

For complementation of the *pfp1‐3*, the wild‐type full coding sequences (CDS) of *PFP1* were cloned into binary vector pCUbi1390 under the control of the maize ubiquitin promoter to produce the recombinant cassette pCUbi1390‐PFP1. The plasmid was then transformed into *agrobacterium tumefaciens* strain EHA105 and subsequently transfected into immature embryo by *Agrobacterium*‐mediated transformation as described previously (Hiei *et al*., 1994).

### Subcellular localization

The coding region of *PFP1* was cloned into the vector pEG101 to generate C‐terminal YFP fusion proteins. Recombinant plasmids were expressed in 3‐week‐old *N. benthamiana* leaves by *Agrobacterium* infiltration, and their signals in plant cells were examined using a Leica SP5 Laser Scanning Confocal Microscope (Leica Microsystems) 48 h after infiltration.

### Protein extraction and Western blot analysis

Protein extraction was conducted in extraction buffer consisting of 50 mm Tris/HCl, pH 8.0, 0.25 m sucrose, 2 mm DTT, 2 mm EDTA and 1 mm phenylmethylsulphonyl fluoride (PMSF; Han *et al*., 2012). Proteins were resolved by SDS‐PAGE and transferred electrophoretically to polyvinylidene difluoride (PVDF) membrane (0.22 μm; GE Healthcare). The membrane was then incubated with specific antibodies and envisioned using Supersignal Chemiluminescent substrates reagent (Thermo Fisher Scientific, Waltham, MA).

### Analysis of starch isolation and properties

Rice grains harvested from *pfp1‐3* and wild‐type mature plants were treated using dehullers and ground into fine flour with a miller. Starch was extracted from polished rice grains through wet milling and alkaline protease. Each sample (20 g) was immersed overnight in a sodium metabisulfite solution (60 mL 0.45%, w:v) in a refrigerator. The solution‐soaked grains were ground using a commercial food blender for 5 min, followed by centrifuged at 2500 ***g*** for 20 min. The supernatant was discarded, and then, the residue was mixed with 0.3 mL alkaline protease by the conditions of enzymatic hydrolysis pH 9, 45°C for 1 h. The treated starch was rinsed three times with deionized water and two times with anhydrous ethanol, dried at 40 °C for 12–48 h.

The starch content of the rice flour was determined with a starch assay kit (Megazyme, Wicklow, Ireland, http://www.megazyme.com/), according to the manufacturer's protocol. Amylose content was evaluated by the method described by Han *et al*. (2012), and that of lipids and proteins by the method described by Kang *et al*. (2005).

The crystalline structure of starch was performed using an X‐ray powder diffractometer (D8, Bruker, Karlsruhe, Germany) with Cu Kα radiation (*λ* = 0.15406 nm) operating at 40 kV and 40 m. The scattered radiation was documented by a NaI crystal scintillation counter in the angular range of 3–40° with a step of 0.02° and a scanning speed of 1°/min. Relative crystallinity (RC) was measured by the peak area of crystallinity as described by Chen *et al*. (2016).

The molecular size and chain length distribution (CLD) of starch was determined by size exclusion chromatography (SEC; Kuang *et al*., 2016). The thermal properties of native starch were determined by a modulated differential scanning calorimeter MDSC 2920 instrument (TA Instruments Inc., Delaware) as previously described with slight modifications (Zhang *et al*., 2018a). The *in vitro* digestion of starch was performed via a three‐stage digestion procedure (Chen *et al*., 2016).

### PFP1 enzyme activity assay

PFP activity in the young leaves of the mutant and wild‐type plants was determined in the glycolytic direction by inspecting the formation of NAD+ as previously described (Lim *et al*., 2009). The enzyme activity assay was measured at 25 °C, and one unit of PFP activity was counted as the formation of 1 μmol of NAD+ per minute.

### RNA isolation, RT‐PCR and qRT‐PCR

Total RNA was extracted from different tissues (root, stem, leaf and developing seeds aged 3, 6 and 12DAF) using an RNAprep pure Plant kit (Invitrogen). An 1 μg aliquot of total RNA was reverse transcribed by priming with oligo (dT18) in a 20 μL reaction volume based on the PrimeScript Reverse Transcriptase kit (Takara, Otsu, Japan). The rice *Actin1* gene used as an internal control of RT‐PCR, and the rice *Ubiquitin* gene as an internal control of qRT‐PCR. qRT‐PCRs were performed as previously described (Qiao *et al*., 2015).

### RNA sequencing (RNA‐Seq) analysis

RNA‐Seq libraries were constructed according to the manufacturer's procedure. An illumina HiSeqTM 2500 was used as a platform for RNA‐Seq via Beijing Novogene Bioinformatics Technology Co. Ltd. Raw RNA‐Seq reads were assessed for quality control by software Trimmomatic v0.32; then, clean reads were aligned to the Nipponbare variety reference genome (http://rice.plantbiology.msu.edu/). The gene expression value was determined by comparing the FPKM value to that in control sample. The genes with more than twofold change and adjusted *P* < 0.005 were considered as differentially expressed genes (DEGs) by DESeq 2 package (bioconductor.org/).

### BiFC and Co‐IP assay

To generate the constructs for BiFC, the full‐length cDNA sequences without a stop codon of LOC_Os06g13810 (OsPFP1β), LOC_Os02g48360 (OsPFP1α1), LOC_Os06g22060 (OsPFP1α2), LOC_Os08g25720 (OsPFP1α3) and LOC_Os09g12650 (OsPFP1α4) were PCR‐amplified using Pfu polymerase, respectively (NEB). For BiFC assay, the PCR products were cloned into the pQBV3 vector and then re‐combined into the pEarleyGate201‐YN and pEarleyGate202‐YC vectors (Lu *et al*., 2010). The resulting constructs were transformed into the Agrobacterium GV3101 and then were transiently expressed in *N. benthamiana* leaves following the method described by Qiao *et al*. (2015). A confocal laser scanning microscope (Zeiss LSM710) was used to monitor YFP fluorescent signals after 48 h post‐infiltration. For Co‐IP assay, the PCR products were cloned into the pQBV3 and pQBV3‐3xFLAG vector and then re‐combined into the pEarleyGate100 and pEarleyGate101 vectors. PFP1β‐3 × FLAG was transiently expressed in *N. benthamiana* together with PFP1α‐YFP‐HA, PFP1α2‐YFP‐HA, PFP1α3‐YFP‐HA and PFP1α4‐YFP‐HA by *Agro*‐infiltration. Total proteins were extracted using an IP buffer [10% (vol/vol) glycerol, 25 mm Tris, pH 7.5, 1 mm EDTA, 150 mm NaCl, 10 mm DTT, 2% (wt/vol) PVPP, 1 × protease inhibitor mixture (Roche), 1 mm PMSF and 0.15% Nonidet P‐40] (Qiao *et al*., 2015), and then incubated with anti‐FLAG affinity gel (Sigma‐Aldrich) or GFP magnetic beads (MBL, Japan) at 4 °C. Coprecipitation signal of PFP1 subunits was detected by using an anti‐FLAG or anti HA antibody (MBL, Japan).

### Determination of metabolites

Soluble sugars were extracted by shaking vigorously in 80% (v/v) ethanol twice after grinding grain endosperm, and the samples were centrifuged at 12 000 ***g*** for 15 min. The supernatant was added chloroform (v/v) for removing the pigment, the solution was centrifuged, and the supernatant was collected. Cell extracts were analysed by ultrahigh‐performance liquid chromatography (Acquity, Waters) coupled to a Q Exactive hybrid quadrupole–orbitrap mass spectrometer (Thermo Fisher Scientific). The injection volume was 10 μL. Metabolites were separated with a Luna NH2 column (100 mm × 2 mm, 3 μm particle size, Phenomenex). The LC‐MS data were analysed as described previously (Zhang *et al*., 2018b). Lipids were extracted from dehulled rice seeds and analysed by liquid chromatography–electrospray ionization mass spectrometry (LC‐SI‐MS). Polar lipids were analysed using an Exion UPLC system coupled with a triple quadrupole/ion trap mass spectrometer (6500 Plus Qtrap; SCIEX). MRM transitions were set up for comparative analysis of various polar lipids (Lim *et al*., 2014). Lipid species in each class were quantified by referencing to spiked internal standards corrected by response factor determined by two standards of same class as described previously (Lu *et al*., 2018).

## Conflict of interest

The authors declare no conflict of interest.

## Author contributions

YQ conceived the study. CC, BH, ZF and YQ designed the experiment. CC, BH, XL, XM, YL, HYY, PZ, HJK, CY and HWX performed the experiment. CC, JY, XW, HYY and YQ analysed the data. YQ wrote the manuscript. All authors read and approved the final manuscript.

## Supporting information


**Figure S1** Agronomic traits of wild‐type and *pfp1‐3* mutant rice plants.
**Figure S2** Confirmation of splice variants in the *pfp1‐3* mutant.
**Figure S3** Relative expression of four *PFP1* genes in *pfp1‐3* mutant and wild‐type plants using quantitative reverse‐transcription PCR (qRT‐PCR).
**Figure S4** Subcellular localization of PFP1‐GFP proteins.
**Figure S5** Analysis of interactions among various PFP1 subunits using bimolecular fluorescence complementation (BiFC) assay.
**Figure S6** Expression analysis of genes involved in the glycolytic pathway and starch biosynthesis in the wild type and pfp1‐3 mutant using RNA‐Seq data.
**Figure S7** Changes of four Phospholipids were determined from individual molecular species in mature rice seeds as revealed by LC‐SI‐MS.

**Table S1** Genetic analysis of the *pfp1‐3* mutant in F_2_ populations.
**Table S2** List of primers used in this study.
**Table S3** List of 16 predicted genes present in candidate region on chromosome 6.
**Table S4** List of rice varieties analyzed using a derived cleaved amplified polymorphic sequence (dCAPS) marker.
**Table S5** Thermal characteristics of the wild type and *pfp1‐3* mutant.
**Table S6** Parameters extracted from the LOS plot and digestogram.
**Table S7** Summary of RNA‐Seq read mapping results.
**Table S8** Transcriptome data of the wild type and *pfp1‐3* mutant.
