# Supplementary material for: Pyrophosphate‐fructose 6‐phosphate 1‐phosphotransferase (PFP1) regulates starch biosynthesis and seed development via heterotetramer formation in rice (Oryza sativa L.)
Source: Plant Biotechnol J. 2019 Jun 14;18(1):83–95. doi: 10.1111/pbi.13173 (PMC6920184; doi:10.1111/pbi.13173)
Supplement: Supplementary file 1 — Figure S1 Agronomic traits of wild‐type and pfp1‐3 mutant rice plants. Figure S2 Confirmation of splice variants in the pfp1‐3 mutant. Figure S3 Relative expression of four PFP1 genes in pfp1‐3 mutant and wild‐type plants using quantitative reverse‐transcription PCR (qRT‐PCR). Figure S4 Subcellular localization of PFP1‐GFP proteins. Figure S5 Analysis of interactions among various PFP1 subunits using bimolecular fluorescence complementation (BiFC) assay. Figure S6 Expression analysis of genes involved in the glycolytic pathway and starch biosynthesis in the wild type and pfp1‐3 mutant using RNA‐Seq data. Figure S7 Changes of four Phospholipids were determined from individual molecular species in mature rice seeds as revealed by LC‐SI‐MS. [file PBI-18-83-s002.pdf]

Supporting Information

Pyrophosphate-fructose 6-phosphate 1-phosphotransferase (PFP1) regulates starch biosynthesis and seed development via heterotetramer formation in rice (*Oryza sativa* L.)

Chen Chen[1,2,#], Bingshu He[2,3,#], Xingxun Liu[4,#], Xiaoding Ma[5], Yujie Liu[6], Hong-Yan Yao[7,8], Peng Zhang[1,2], Junliang Yin[1], Xin Wei[2], Hee-Jong Koh[9],Chen Yang[6], Hong-Wei Xue[7,9], Zhengwu Fang[1*], Yongli Qiao[2 *]

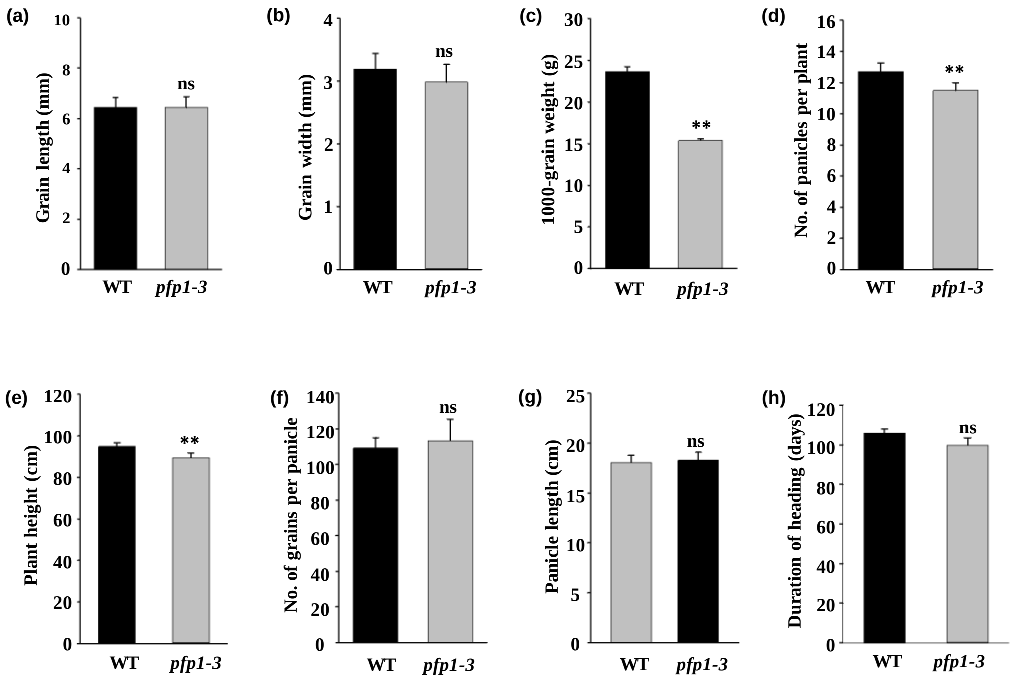

Figure S1. Agronomic traits of wild-type and *pfp1-3* mutant rice plants.

(a) Grain length. (b) Grain width. (c) 1000-grain weight. (d) Number of panicles per plant. (e) Plant height. (f) Number of grains per panicle. (g) Panicle length. (h) Duration to heading. Wild-type cultivar Hwacheong (WT) and *pfp1-3* mutant plants were grown under natural paddy field conditions. Data represent mean ± standard error (SE; $n = 15$). Asterisks indicate significant differences between wild-type and *pfp1-3* mutant plants (**, $P < 0.01$; Welch's t-test). ns, not significant.

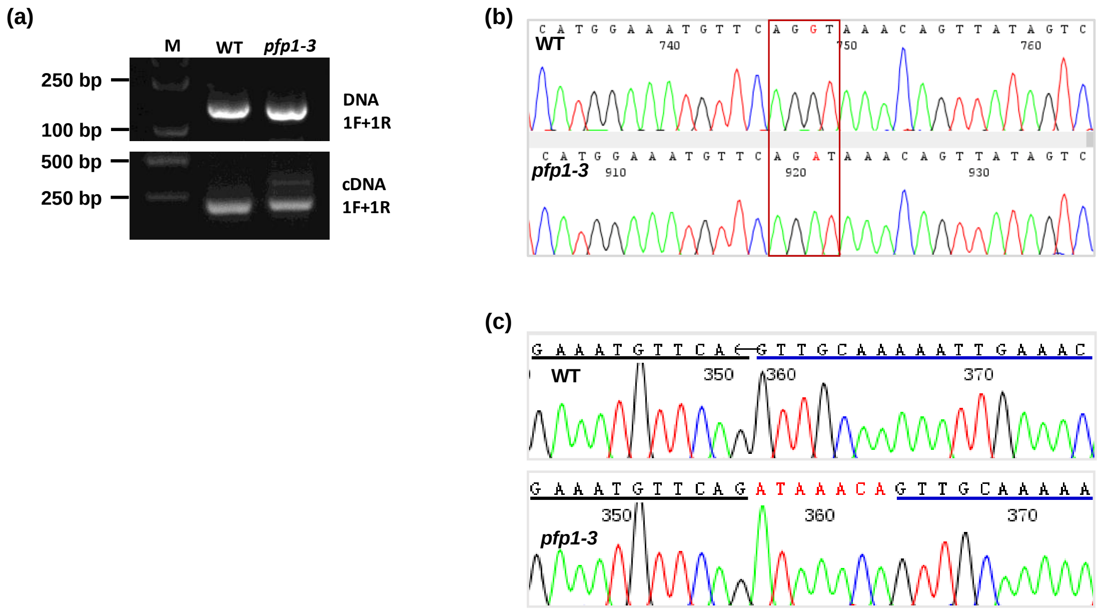

Figure S2. Confirmation of splice variants in the *pfp1-3* mutant.

(a) PCR-based confirmation of the deletion of a genomic fragment using two sequence-specific primer pairs. PCR primers were located within the deleted regions and/or coding regions. The amplified products were also confirmed by sequencing. (b, c) Chromatograms showing the sequence of PCR products amplified from genomic DNA (b) and cDNA (c) of *pfp1-3* and wild-type plants (WT).

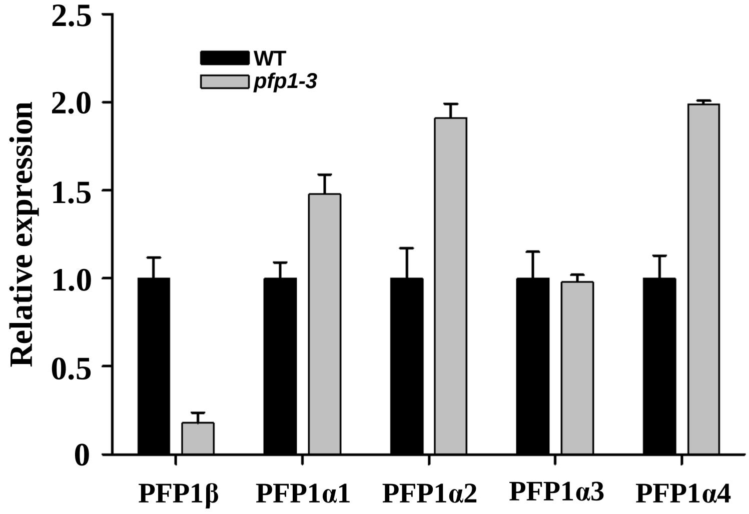

Figure S3. Relative expression of four *PFP1* genes in *pfp1-3* mutant and wild-type plants using quantitative reverse-transcription PCR (qRT-PCR).

Total RNA was isolated from endosperm of *pfp1-3* mutant and wild-type plants at 6 days after pollination (DAP). Data represent mean ± SD of three biological replicates.

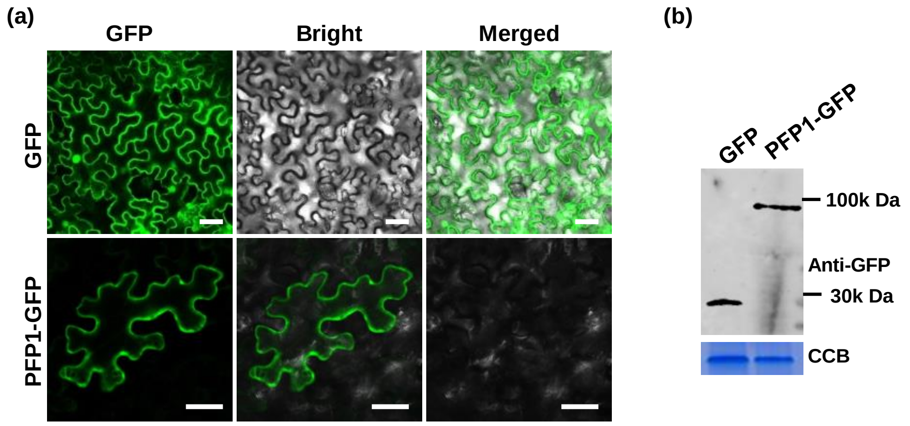

Figure S4. Subcellular localization of PFP1-GFP proteins.

(a) Confocal micrographs of *Nicotiana benthamiana* leaves expressing *PFP1-GFP*. (b) Immunodetection of PFP1 protein using anti-GFP antibody, Coomassie brilliant blue (CBB) staining as a loading control for Western blot analysis. Scale bars, 35 µm.

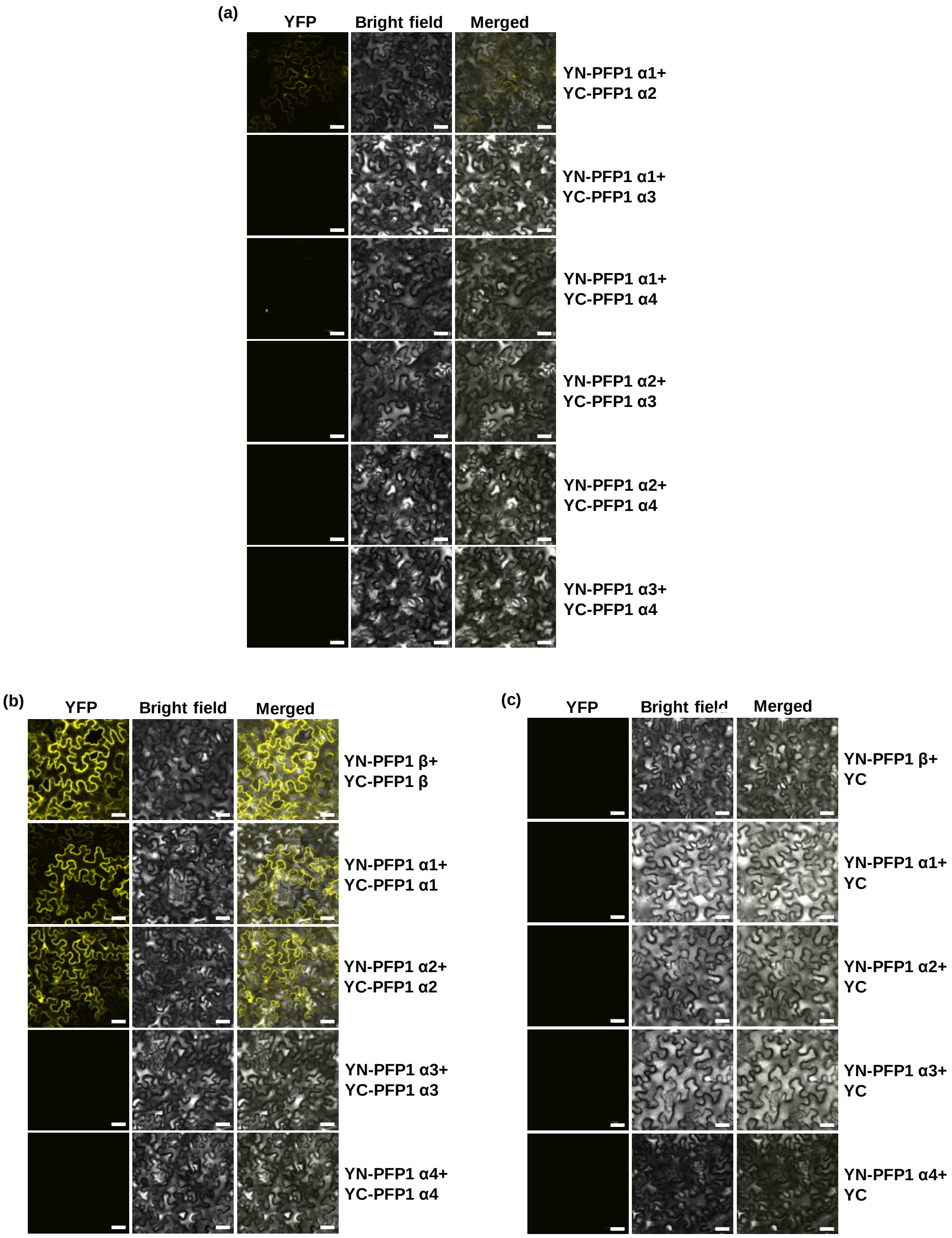

Figure S5. Analysis of interactions between various PFP1 subunits using bimolecular fluorescence complementation (BiFC) assay.

(a) BiFC assay confirming interactions bewteen 4 PFP1α proteins. (b) BiFC assay confirming homodimer formation in 5 PFP1 proteins. (c) BiFC assay confirming autofluorescence among PFP1 subunits. The BiFC assay was performed in *N. benthamiana* leaves upon *Agrobacterium tumefaciens*-mediated transient expression. Fluorescence was detected in epidermal cells of infiltrated tissues by confocal microscopy at 48 hpi. Scale bars, 30 μm.

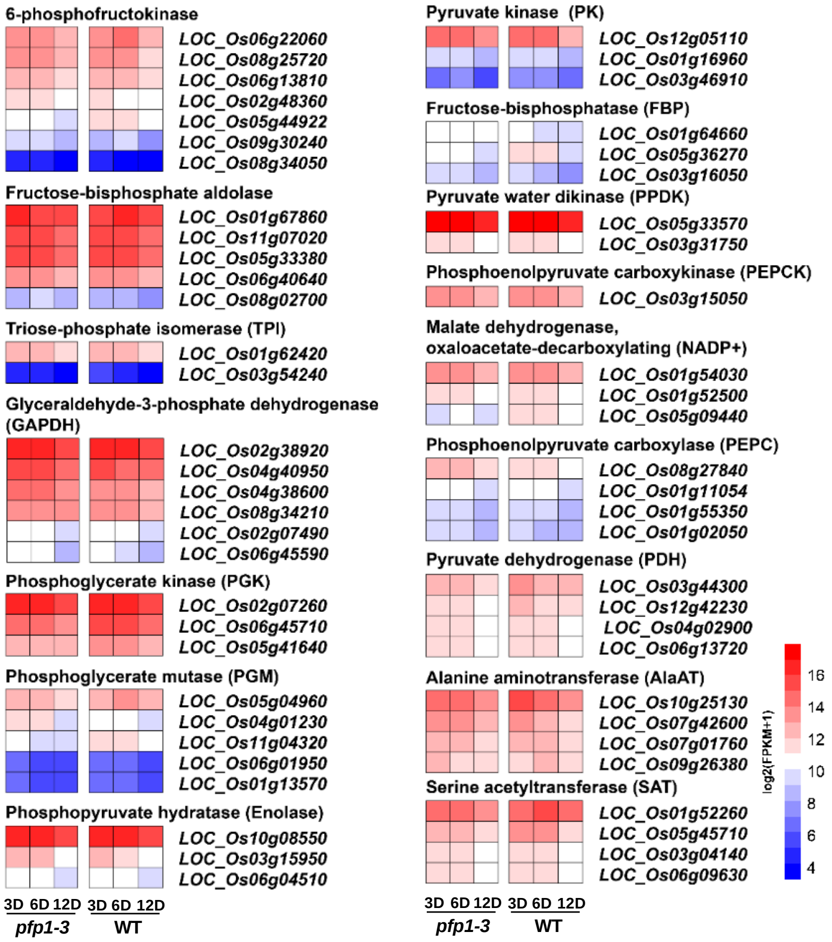

Figure S6. Expression analysis of genes involved in the glycolytic pathway and starch biosynthesis in the wild type and pfp1-3 mutant using RNA-seq data.

Expression profiles of genes involved in glycolysis, pyruvate metabolism, and FA metabolism. Total RNA was isolated from seeds at 3, 6, and 12 DAF, and mRNA subjected to reverse transcription using oligo (dT) primers. Blue and red colors indicate downregulated and upregulated expression levels, respectively, in comparison with expression levels in wild-type rice plants.

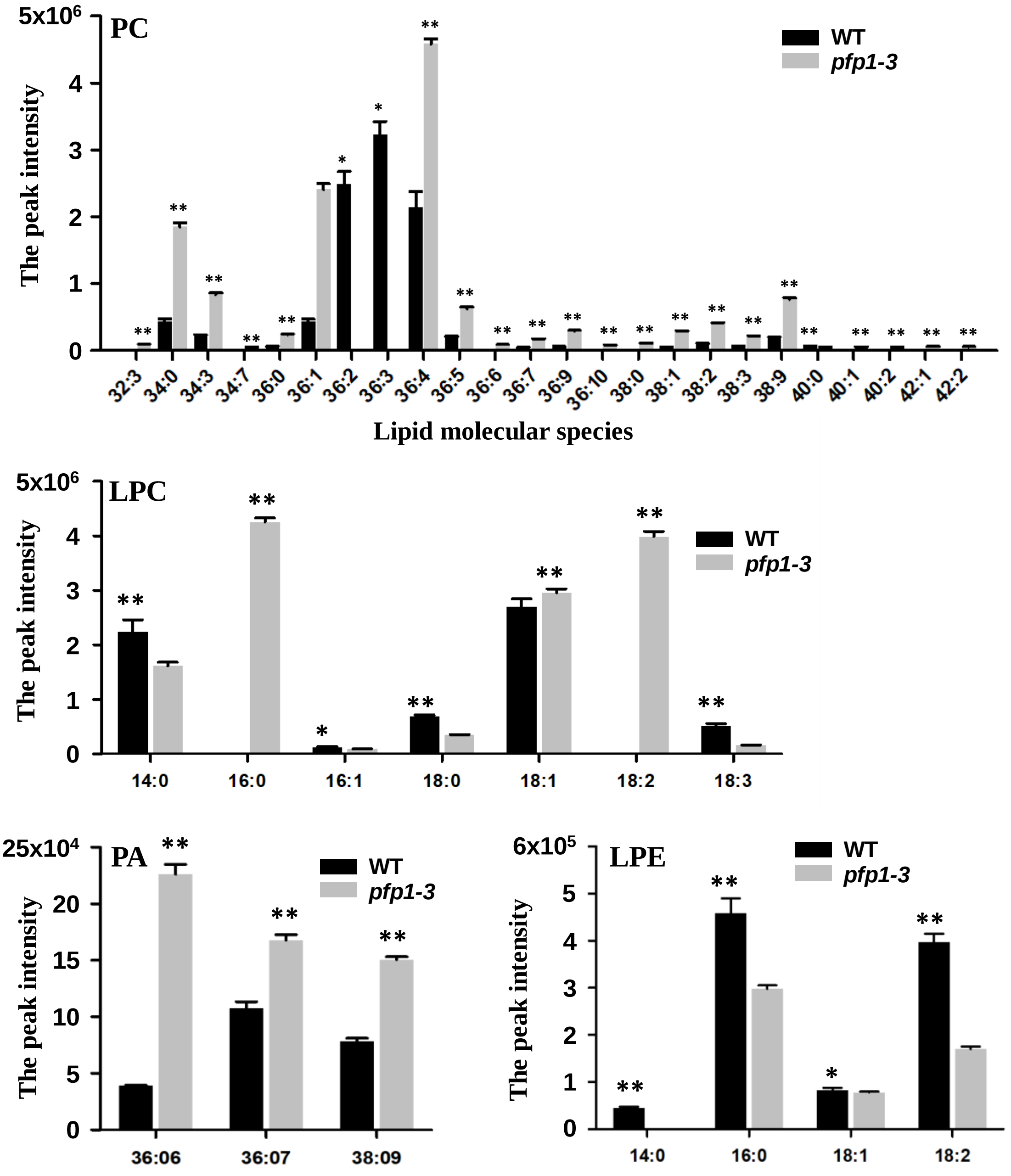

Figure S7. Changes of four Phospholipids were determined from individual molecular species in mature rice seeds as revealed by LC-SI-MS.

The values represent mean ± SE of two biological replicates. Asterisks represent significant differences between the *pfp1-3* mutant and wild type (*, P < 0.05; **, P < 0.01; Student's t-test).

Table S1. Genetic analysis of the *pfp1-3* mutant in F_2 populations.

Cross	Number of floury endosperms	Number of normal endosperms	χ^2
pfp1-3 × Dular	554	1610	0.4165
pfp1-3 × ZH11	317	949	0.0011

Table S2. List of primers used in this study.

	Primer name	Primer sequence (5' to 3')
SSR, InDel and STS markers for mapping	RM4128-F	AGTAACTCGATCAAACTAAC
	RM4128-R	AGAGTCCATATAGAATTTCA
	C6-5-F	GCTTCTCCCGGAGTATGTCA
	C6-5-R	TGGTCTGAAAAGTGCCAAAA
	P20-F	CATCAACCCAAGCCAACCAA
	P20-R	TCGTTTCGGCTAGGTGAAGT
	P23-F	TCAGGCCAACGATGAGGTT
	P23-R	TTGCACCCACTGTTAGCTCT
	Q3-F	CAGTCCTAGATTCATTTTCTAG
	Q3-R	TTGGTTCTTGCATGTCTTTGGAG
	P40-F	CGACACAGTCTCTAAAACACAACTT
	P40-R	TCACAAGGTATGTAGTTAAAACTAA
	P32-F	GGTACGTGTTGATTCGGCAA
	P32-R	AAATTAAATTATTCGGAATCG
	Q10-F	CGTGACCAAGAAAAGAAAAAACTAA
	Q10-R	TAGTACTGACCTACTCCTAGCACTA
	P22-F	GCTTCTGTGCTTGCTTGATT
	P22-R	CCAAGCATTCACACCAACCA
	P17-F	TGTGCATTTTCTCCTCCTCCT
	P17-R	TGTGCATTTTCTCCTCCTCCT
Transgenic plants	p1302-PFP1-F	CTTGACCATGGTAGATCTGACTAGTATGGCGGCGGCGGCGGTGGCG
	p1302-PFP1-R	TGAAAAGTTCTTCTCCTTTACTAGTTGCCTCGGCGCCGAGTTCCAAC
BiFC and Co-IP entry vector	QBV3-PFP1-F	AAAAAAGCAGGCTCAGGGGATATCATGGCGGCGGCGGCGGTGGCG
	QBV3-PFP1-R	GAAAGCTGGGTGCAGGGCGATATCTGCCTCGGCGCCGAGTT
	QBV3-PFP1-FLAG-R	GTCTTTGTAGTCCTCGACGATATCTGCCTCGGCGCCGAGTTCCAAC
	QBV3-PFP1α1-F	AAAAAAGCAGGCTCAGGGGATATCATGGACTCCGACTACGGCGTG
	QBV3-PFP1α1-R	GAAAGCTGGGTGCAGGGCGATATCGAGTGGGGTTCCCTGGC
	QBV3-PFP1α2-F	AAAAAAGCAGGCTCAGGGGATATCATGGGCAGCGTGGCGATGGAC
	QBV3-PFP1α2-R	GAAAGCTGGGTGCAGGGCGATATCGATGGTTGCTTGGCCAC
	QBV3-PFP1α3-F	AAAAAAGCAGGCTCAGGGGATATCATGAACGCCGACTTCGGCGCG
	QBV3-PFP1α3-R	GAAAGCTGGGTGCAGGGCGATATCGTTGAAATGGTAGAGCG
	QBV3-PFP1α4-F	AAAAAAGCAGGCTCAGGGGATATCATGTCCATGAACGCGGACCTC
	QBV3-PFP1α4-R	GAAAGCTGGGTGCAGGGCGATATCGTTCAGATTGAACAGAG
qRT-PCR	PFP1-RT-F	GAGGCACGGAAAGTACAAGC
	PFP1-RT-R	GTCACTCTGCGAGGTTCCAT
	PFP1α1-RT-F	TGACATCGTCTTCCACTGGC
	PFP1α1-RT-R	CGAAAAAGAAAGCCGTGGGG
	PFP1α2-RT-F	TCCCTCTTTCAGTGGCCAAG
	PFP1α2-RT-R	CACAAACCACTCGCAATGCA
	PFP1α3-RT-F	TCTACAGGACTCCAGGAGGC

	PFP1α3-RT-R	AGCATCCAGGCTTCACCATC
	PFP1α4-RT-F	GGAACATTGTGAAGCCCGGA
	PFP1α4-RT-R	CTCTGCATAGAAGGGAGCCG
	OsActin-F	CTGCGATAATGGAACTGGT
	OsActin-R	ACAATGCTGGGGAAGACA
Confirming point mutation	1-F	AGCTTCAACCAGAATCCAGG
	1-R	CTGAAATGTGCGGGGTACTT
	dCAPS-F	GTTTGACAATCTTCATTCACCTGTG (*EcoRV*)
	dCAPS-R	GCGAAGGGAGGACTATAACTGTTTA (*EcoRV*)

†SSR, simple sequence repeat; InDel, insertion/deletion; STS, sequence tagged site. dCAPS, derived cleaved amplified polymorphic sequence.

Table S3. List of 16 predicted genes present in candidate region on chromosome 6.

ORF[†]	Locus	Gene annotation
1	Os06g13670	E2F family transcription factor protein, putative, expressed
2	Os06g13680	B12D protein, putative, expressed
3	Os06g13690	Expressed protein
4	Os06g13700	Hypothetical protein
5	Os06g13710	Glycosyltransferase, putative, expressed
6	Os06g13720	Dehydrogenase E1 component domain containing protein, expressed
7	Os06g13730	Glutamate receptor precursor, putative, expressed
8	Os06g13740	Transposon protein, putative, unclassified, expressed
9	Os06g13750	Expressed protein
10	Os06g13760	Glycosyl transferase 8 domain containing protein, putative, expressed
11	Os06g13770	Transposon protein, putative, unclassified
12	Os06g13780	Expressed protein
13	Os06g13800	Expressed protein
**14**	**Os06g13810**	**Pyrophosphate-fructose 6-phosphate**
15	Os06g13820	Dynamin, putative, expressed
16	Os06g13830	Endoglucanase, putative, expressed

[†]ORF, open reading frame.

Table S4. List of rice varieties analyzed using a derived cleaved amplified polymorphic sequence (dCAPS) marker.

No	Variety	Origin	Type	No.	Variety	Origin	Type
1	*pfp1-3*	Mutant	Japonica	10	Yuewanxian 3	China	Indica
2	Hwacheong	Korea	Japonica	11	IR64	Philippines	Indica
3	WY1	China	Japonica	12	9311	China	Indica
4	Guichao 2	China	Japonica	13	Milyang 23	Korea	Indica
5	Huaidao 5	China	Japonica	14	N22	India	Indica
6	Jigeng 80	China	Japonica	15	12-927	China	Indica
7	Tong 35	China	Japonica	16	Dular	India	Indica
8	Zhonghua11	China	Japonica	17	ZD05348	China	Indica
9	Nipponbare	Japan	Japonica				

Table S5. Thermal characteristics of the wild type and *pfp1-3* mutant.

Genotype	T_O (°C)	T_P (°C)	T_C (°C)	ΔH (J/g)
Wild type	61.20 ± 0.30a	65.50 ± 0.20b	71.60 ± 0.15a	11.64 ± 0.31a
pfp1-3 mutant	57.30 ± 0.40b	66.45 ± 0.05a	71.90 ± 0.20a	11.47 ± 0.44a

Data represent ± standard deviation (SD). T_O, onset temperature; T_P, peak temperature; T_C, conclusion temperature; ΔH, gelatinization enthalpy. Different letters in the same column represent significant difference at $p < 0.01$.

Table S6. Parameters extracted from the LOS plot and digestogram.

Code	Genotype	C∞ (%)	K (10^{-2} min^{-1})
1	*pfp1-3* mutant	84.00 ± 1.87a	2.678 ± 0.239a
2	Wild type	78.01 ± 0.16b	2.335 ± 0.174a

Data represent mean ± standard error (SE). Different letters in the same column represent significant differences ($P < 0.01$). K, digestion rate; C∞, product concentration at the end of the reaction.

Table S7. Summary of RNA-seq read mapping results

Samples	Time	Replicates	Total reads	Mapped reads (%)	Unique (%)	Non specifically (%)
pfp1-3	3D	rep1	37280754	31263762 (83.86%)	28680601 (76.93%)	2583161 (6.93%)
		rep2	39758138	31467850 (79.15%)	28363306 (71.34%)	3104544 (7.81%)
	6D	rep1	25793068	20770555 (80.53%)	16968996 (65.79%)	3801559 (14.74%)
		rep2	59091926	47605748 (80.56%)	42919935 (72.63%)	4685813 (7.93%)
	12D	rep1	50153020	39521474 (78.80%)	27986737 (55.80%)	11534737 (23.00%)
		rep2	43060102	32999252 (76.64%)	19960799 (46.36%)	13038453 (30.28%)
WT	3D	rep1	26881962	24746840 (92.06%)	21857622 (81.31%)	2889218 (10.75%)
		rep2	26861458	20235672 (75.33%)	17683091 (65.83%)	2552581 (9.50%)
	6D	rep1	42002148	34858158 (82.99%)	26572627 (63.26%)	82855311 (9.73%)
		rep2	44729450	41235400 (92.19%)	36867579 (82.42%)	4367821 (9.76%)
	12D	rep1	43820522	33824704 (77.19%)	30105233 (68.70%)	3719471 (8.49%)
		rep2	41573972	32646300 (78.53%)	29466818 (70.88%)	3179482 (7.65%)
